# Crossing the Septum: May-Thurner Syndrome and Patent Foramen Ovale as a Dual Mechanism for Recurrent Ischemic Stroke

**DOI:** 10.7759/cureus.110041

**Published:** 2026-06-01

**Authors:** Irina Ter-Ovanesyan, Daniela Prado Escobar, Kian Memari, Anastasiya Sizova, Josue P Boutros, Shane Williams, Peter Cohen, Lissette P Lazo

**Affiliations:** 1 Medical School, Nova Southeastern University Dr. Kiran C. Patel College of Osteopathic Medicine, Fort Lauderdale, USA; 2 Internal Medicine, Nova Southeastern University Dr. Kiran C. Patel College of Osteopathic Medicine, Clearwater, USA; 3 Family Medicine, Palmetto General Hospital, Hialeah, USA; 4 Family Medicine, Nova Southeastern University Dr. Kiran C. Patel College of Osteopathic Medicine, Fort Lauderdale, USA

**Keywords:** cryptogenic stroke, iliac vein compression, may-thurner syndrome, paradoxical embolism, patent foramen ovale, recurrent ischemic stroke, venoplasty

## Abstract

May-Thurner syndrome (MTS) is an anatomic venous compression disorder in which the left common iliac vein is compressed by the overlying right common iliac artery, predisposing patients to venous stasis and thrombosis. A patent foramen ovale (PFO) is a persistent interatrial communication that may permit paradoxical embolization when right-to-left shunting occurs. Although each condition is independently recognized, their coexistence as a potential mechanism for recurrent ischemic stroke is uncommon and may be overlooked when routine lower-extremity venous imaging is unrevealing. We present the case of a 70-year-old female with prior ischemic stroke, pulmonary embolism, and deep vein thrombosis (DVT) on chronic apixaban who presented with two days of isolated left upper extremity weakness. Brain magnetic resonance imaging (MRI) demonstrated an acute ischemic infarct in the right high frontal lobe involving the precentral gyrus. Bilateral lower-extremity venous duplex ultrasound was negative for acute DVT and did not demonstrate definite chronic post-thrombotic changes in the visualized femoropopliteal segments; however, this did not exclude proximal iliac venous pathology. Transesophageal echocardiography (TEE) demonstrated a PFO with right-to-left shunting. Pelvic magnetic resonance angiography demonstrated narrowing of the left common iliac vein from extrinsic arterial compression, consistent with MTS. The patient underwent balloon venoplasty of the left iliac vein and was referred for outpatient PFO closure evaluation. This case highlights the importance of expanded venous and cardiac evaluation in selected patients with recurrent or cryptogenic stroke and prior venous thromboembolism.

## Introduction

May-Thurner syndrome (MTS), also known as iliac vein compression syndrome, occurs when the left common iliac vein is compressed between the overlying right common iliac artery and the lumbar spine. This compression may promote venous stasis, endothelial injury, and thrombosis, most commonly involving the left iliofemoral venous system [1]. Although classically described in younger women with unexplained left-sided deep vein thrombosis (DVT), clinically significant iliac vein compression may be underrecognized outside the typical demographic profile [2].

A patent foramen ovale (PFO) is present in approximately one-quarter of adults and is usually clinically silent. In selected patients, however, a PFO may provide a conduit for paradoxical embolism, allowing venous thrombi to bypass pulmonary filtration and enter the arterial circulation [3]. PFO-associated stroke remains diagnostically nuanced because PFOs are common and may be incidental, particularly in older patients with competing vascular risk factors [4].

The coexistence of MTS and PFOs creates a plausible but uncommon pathway for ischemic stroke: venous thrombus formation or venous stasis from iliac compression followed by systemic embolization through a right-to-left interatrial shunt. This mechanism may be missed when evaluation stops after negative lower-extremity duplex ultrasound, which has limited ability to assess pelvic and iliac veins. We present a case of recurrent ischemic stroke in an older patient with prior venous thromboembolism who was ultimately found to have both MTS and PFOs.

## Case presentation

A 70-year-old female with a history of ischemic stroke with residual right facial droop, pulmonary embolism, and DVT on chronic apixaban presented with two days of isolated left upper extremity weakness. She denied recent trauma, headache, dizziness, visual disturbance, dysarthria, aphasia, or other new focal neurologic symptoms (Table 1). 

**Table 1:** Timeline from symptom onset to diagnosis and intervention CT: computed tomography; MRI: magnetic resonance imaging; DVT: deep vein thrombosis; TEE: transesophageal echocardiography; PFO: patent foramen ovale; MRA: magnetic resonance angiography; MTS: May-Thurner syndrome

Time point	Event
Prior history	Ischemic stroke with residual right facial droop; history of pulmonary embolism and DVT on chronic apixaban
Day -2	Onset of new isolated left upper extremity weakness
Day 0	Emergency evaluation; non-contrast head CT negative for acute hemorrhage
Day 1	Brain MRI demonstrated acute right high frontal/precentral gyrus infarct
Days 1–2	Lower-extremity venous duplex negative for acute DVT and without definite chronic post-thrombotic changes in visualized femoropopliteal segments
Day 2	TEE demonstrated PFO with right-to-left shunting
Days 2–3	Pelvic MRA demonstrated left common iliac vein compression consistent with MTS
Hospital course	Aspirin, high-intensity statin therapy, and apixaban continued
Intervention	Balloon venoplasty of left common iliac vein
Follow-up Plan	Outpatient referral for PFO closure evaluation

Neurologic examination confirmed focal left upper extremity weakness without additional acute cortical findings. Non-contrast CT of the head showed no acute hemorrhage. Brain MRI without intravenous contrast demonstrated an acute ischemic focus in the right high frontal lobe involving the precentral gyrus, corresponding to the patient’s left upper extremity symptoms (Figures 1, 2).

**Figure 1:**
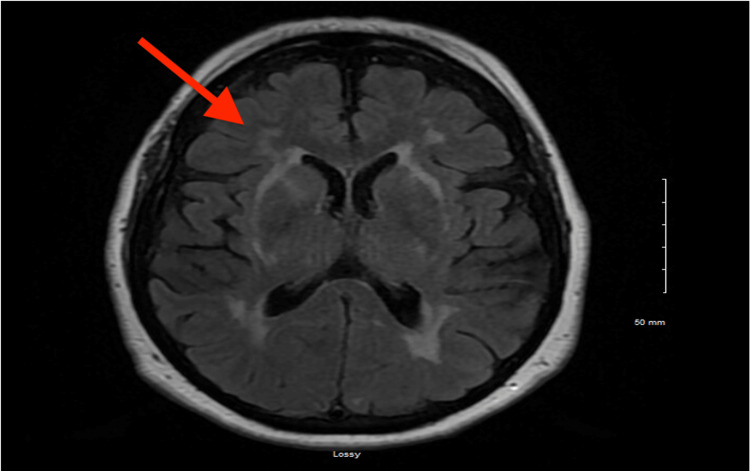
Brain MRI without intravenous contrast demonstrating an acute ischemic focus in the right high frontal lobe involving the precentral gyrus (red arrow) The infarct location corresponds clinically to the patient’s isolated left upper extremity weakness.

**Figure 2:**
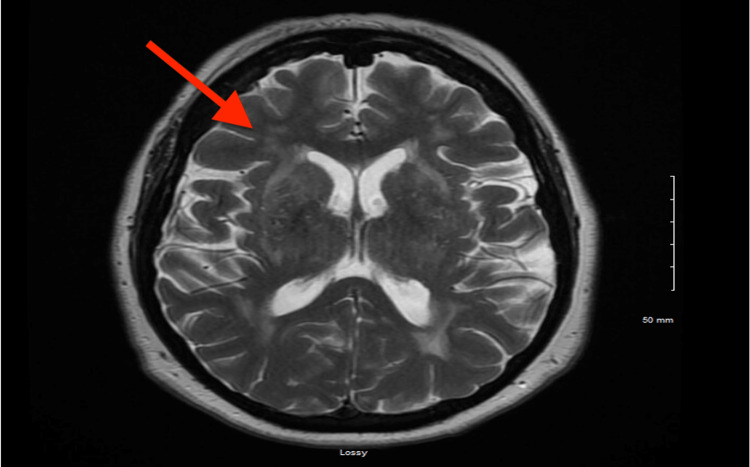
Additional brain MRI image demonstrating the right frontal/precentral ischemic lesion (red arrow) The imaging supports an acute right middle cerebral artery territory infarct.

Given the patient’s history of pulmonary embolism and DVT despite chronic anticoagulation, a paradoxical embolic mechanism was considered. Bilateral lower extremity venous duplex ultrasound did not demonstrate acute DVT. The visualized femoropopliteal venous segments did not show definite chronic thrombotic or post-thrombotic changes, such as persistent noncompressibility, echogenic intraluminal material, venous wall thickening, or collateralized femoropopliteal flow. However, the study did not adequately assess the iliac venous system, and a proximal pelvic venous source could not be excluded.

Transesophageal echocardiography (TEE) demonstrated a PFO with right-to-left shunting (Figure 3). Pelvic MRA with intravenous contrast demonstrated focal narrowing of the left common iliac vein due to compression by the overlying iliac artery, consistent with MTS (Figures 4, 5). Pertinent diagnostic findings are summarized in Table 2.

**Figure 3:**
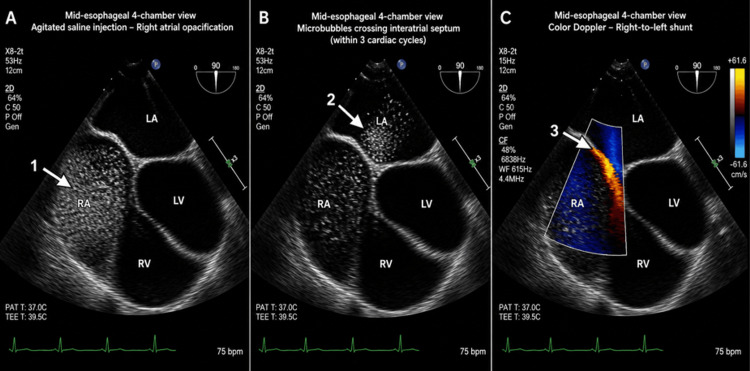
Transesophageal echocardiography demonstrating patent foramen ovale with right-to-left shunting A) TEE bubble study demonstrating right atrial opacification: Mid-esophageal four-chamber transesophageal echocardiography (TEE) view following agitated saline injection demonstrates dense microbubble opacification of the right atrium (arrow 1). No immediate left-sided opacification is seen at the initial phase. B) TEE demonstrating microbubble transit across the interatrial septum: Mid-esophageal four-chamber TEE view demonstrating microbubbles traversing the interatrial septum from the right atrium to the left atrium (arrow 2) within three cardiac cycles of right atrial opacification, consistent with intracardiac right-to-left shunting through a patent foramen ovale. C) Color Doppler imaging demonstrating right-to-left shunt across the PFO: Color Doppler interrogation of the interatrial septum demonstrates right-to-left flow across the patent foramen ovale (arrow 3), further confirming the presence of an interatrial shunt.

**Figure 4:**
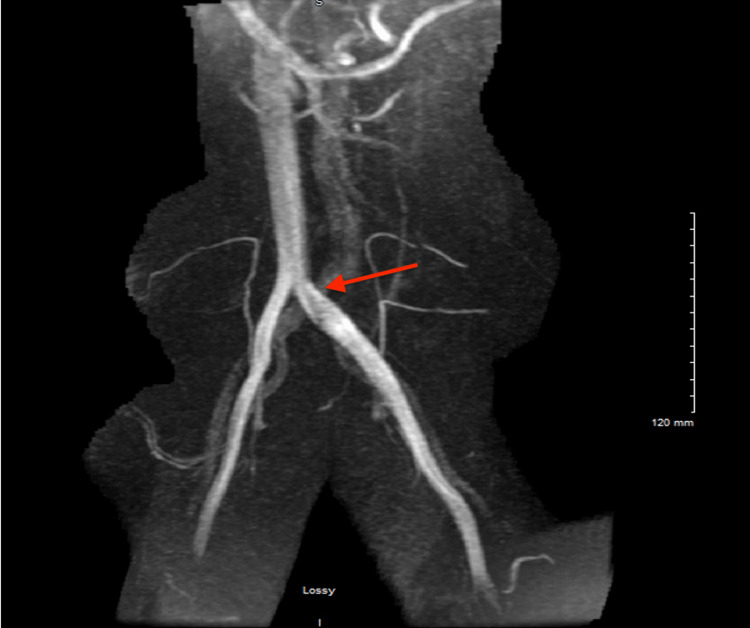
MRA pelvis with intravenous contrast demonstrating narrowing of the left common iliac vein due to compression by the overlying iliac artery (red arrow) This finding is consistent with May-Thurner syndrome and provides an anatomic substrate for venous stasis and thrombus formation.

**Figure 5:**
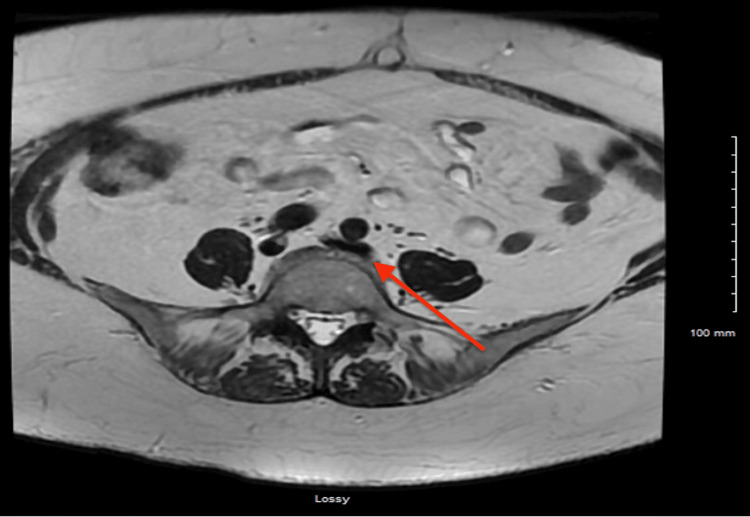
Additional MRA pelvis image demonstrating focal left common iliac vein narrowing at the site of arterial compression (red arrow) The imaging further supports iliac vein compression as a contributor to the patient’s venous thromboembolic history.

**Table 2:** Pertinent diagnostic findings CT: computed tomography; MRI: magnetic resonance imaging; DVT: deep vein thrombosis; TEE: transesophageal echocardiography; PFO: patent foramen ovale; MRA: magnetic resonance angiography; MTS: May-Thurner syndrome

Test or study	Result	Clinical significance
Non-contrast CT head	No acute hemorrhage	Helped exclude intracranial hemorrhage
Brain MRI without IV contrast	Acute ischemic focus in the right high frontal lobe/precentral gyrus	Correlated with the left upper extremity weakness
Bilateral lower extremity venous duplex ultrasound	No acute DVT; no definite chronic post-thrombotic changes in the visualized femoropopliteal veins	Did not exclude iliac vein compression or pelvic venous thrombosis
TEE	PFO with right-to-left shunting	Supported possible paradoxical embolic pathway
Pelvic MRA with IV contrast	Narrowing of the left common iliac vein due to arterial compression	Diagnostic of MTS
Anticoagulation status	Chronic apixaban therapy	Stroke occurred despite chronic anticoagulation

The patient was managed with aspirin, high-intensity statin therapy, and continuation of apixaban. Given the identified iliac venous compression, she underwent balloon venoplasty of the left common iliac vein. Iliac vein stenting was considered but deferred during the index intervention because no acute iliofemoral DVT was identified, the intervention was performed as an initial flow-restoring and diagnostic therapeutic maneuver, and the team favored outpatient reassessment before permanent venous stent placement. She was referred for outpatient PFO closure evaluation as part of secondary stroke prevention planning. Her case was managed through a multidisciplinary approach involving neurology, cardiology, and vascular/interventional specialists.

## Discussion

This case demonstrates a clinically important dual mechanism for recurrent ischemic stroke involving venous thromboembolic predisposition from MTS and a right-to-left embolic conduit through a PFO. Although MTS and PFOs are individually recognized, their coexistence requires deliberate diagnostic consideration because standard stroke evaluations may not routinely assess pelvic venous anatomy.

MTS is traditionally associated with left iliofemoral DVT, but iliac vein compression is not reliably excluded by routine lower-extremity duplex ultrasound. Duplex ultrasound is useful for femoropopliteal DVT evaluation but has reduced sensitivity for pelvic and iliac venous pathology due to anatomic depth, bowel gas, body habitus, and limited acoustic windows [1]. In this patient, the negative duplex study excluded acute DVT in the visualized lower-extremity veins and did not demonstrate definite chronic post-thrombotic femoropopliteal changes, but it did not exclude iliac vein compression or pelvic venous thrombosis. Pelvic MRA was therefore critical in identifying the anatomic substrate for venous stasis.

PFO-associated stroke remains a nuanced diagnosis because a PFO is common in the general population and may be incidental, especially in older patients with competing vascular risk factors [3,4]. However, the likelihood of a clinically relevant paradoxical embolic mechanism increases when a PFO coexists with a history of venous thromboembolism, recurrent ischemic stroke, demonstrable right-to-left shunting, and a structural venous abnormality, such as MTS. In this case, the PFO should be interpreted as a plausible contributor rather than definitive proof of causality. Randomized trials have demonstrated reduced recurrent stroke risk with PFO closure in carefully selected patients with cryptogenic stroke and high-risk PFO features, supporting consideration of closure when paradoxical embolism is strongly suspected [5].

The patient’s stroke occurred despite chronic apixaban therapy. This raises the possibility of residual risk related to mechanical venous stasis from iliac compression, intermittent embolic risk, competing stroke mechanisms, or anticoagulation failure. The available case data did not definitively establish medication nonadherence, inappropriate dosing, renal dysfunction, or drug interaction as the cause of recurrence. Therefore, the mechanism was framed as probable multifactorial risk with paradoxical embolism remaining a leading consideration.

The decision to proceed with balloon venoplasty alone merits clarification. Venous stenting is commonly considered in symptomatic or clinically significant MTS, particularly when fixed iliac vein compression persists or when recurrent iliofemoral thrombosis is present. In this patient, balloon venoplasty was selected as the initial intervention because there was no acute iliofemoral DVT identified, the immediate goal was to improve venous outflow while minimizing the risks of permanent venous stent placement, and multidisciplinary planning included outpatient reassessment. Stenting remained a potential future option if significant residual stenosis, recurrent thrombosis, or persistent symptoms were demonstrated.

The clinical lesson is that recurrent stroke in a patient with prior venous thromboembolism should prompt consideration of both a venous source and an embolic conduit. When routine lower-extremity duplex ultrasound is negative but suspicion remains, pelvic venous imaging and TEE may identify treatable mechanisms that would otherwise be missed.

## Conclusions

This case highlights the coexistence of MTS and PFOs as a plausible dual mechanism for recurrent ischemic stroke. The patient’s history of venous thromboembolism, recurrent cerebral ischemia, PFO with right-to-left shunting, and imaging-confirmed left common iliac vein compression supported paradoxical embolism as an important diagnostic consideration, although definitive proof of causality is inherently difficult in older patients with competing vascular risk factors.

A comprehensive stroke evaluation should extend beyond standard arterial and cardiac testing when clinical history suggests venous thromboembolic risk. Pelvic venous imaging may be particularly valuable when lower-extremity duplex ultrasound is negative but suspicion for proximal venous pathology remains. Management may require pharmacologic therapy, venous intervention, and individualized consideration of PFO closure.
